# Supplementary figures and images for: BMSCs differentiated into neurons, astrocytes and oligodendrocytes alleviated the inflammation and demyelination of EAE mice models
Source: PLoS One. 2021 May 13;16(5):e0243014. doi: 10.1371/journal.pone.0243014 (PMC8118321; doi:10.1371/journal.pone.0243014)

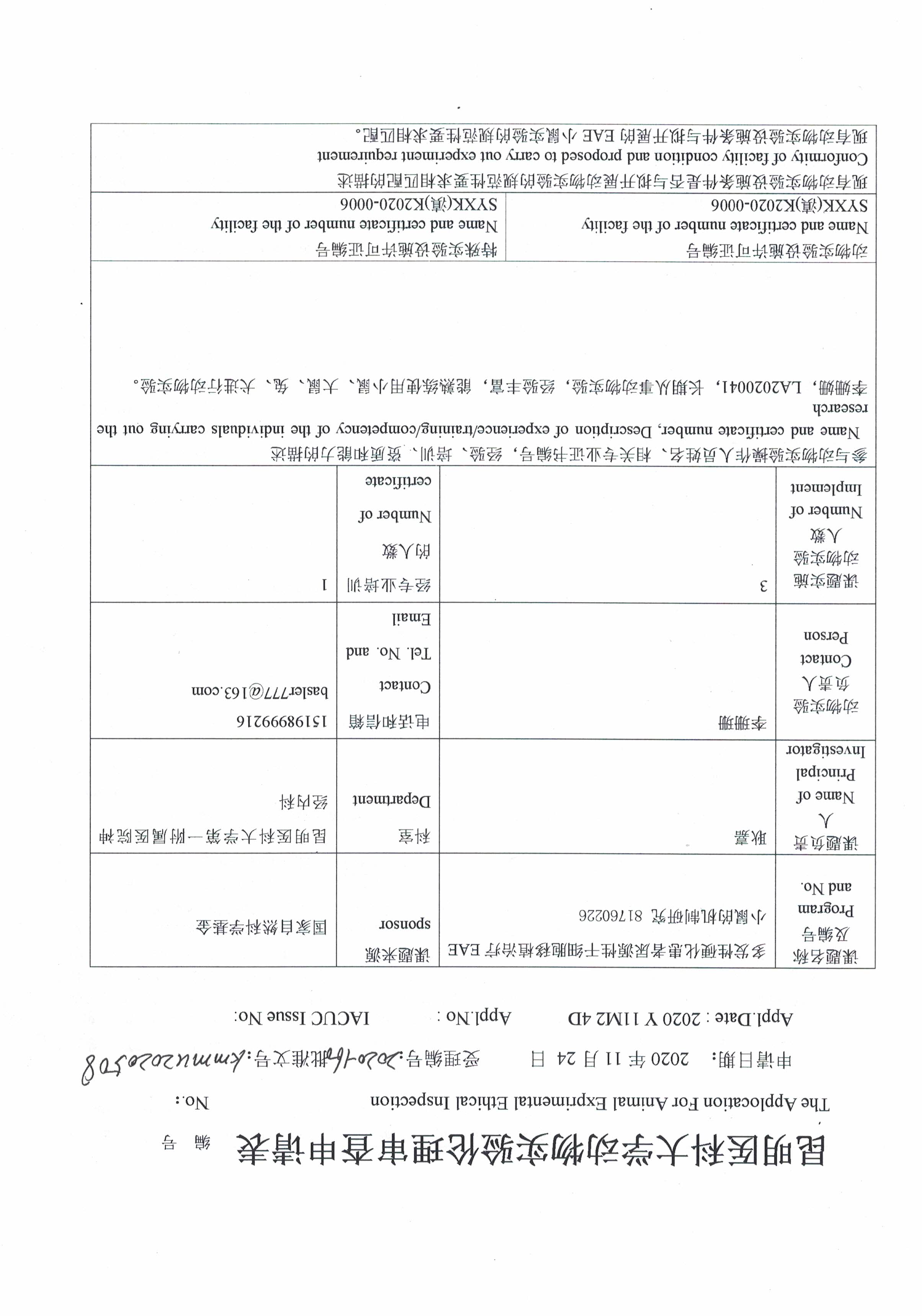

Supplement: S1 File — (ZIP) [file pone.0243014.s002.zip › Ethical approval/CCI_000062.jpg]

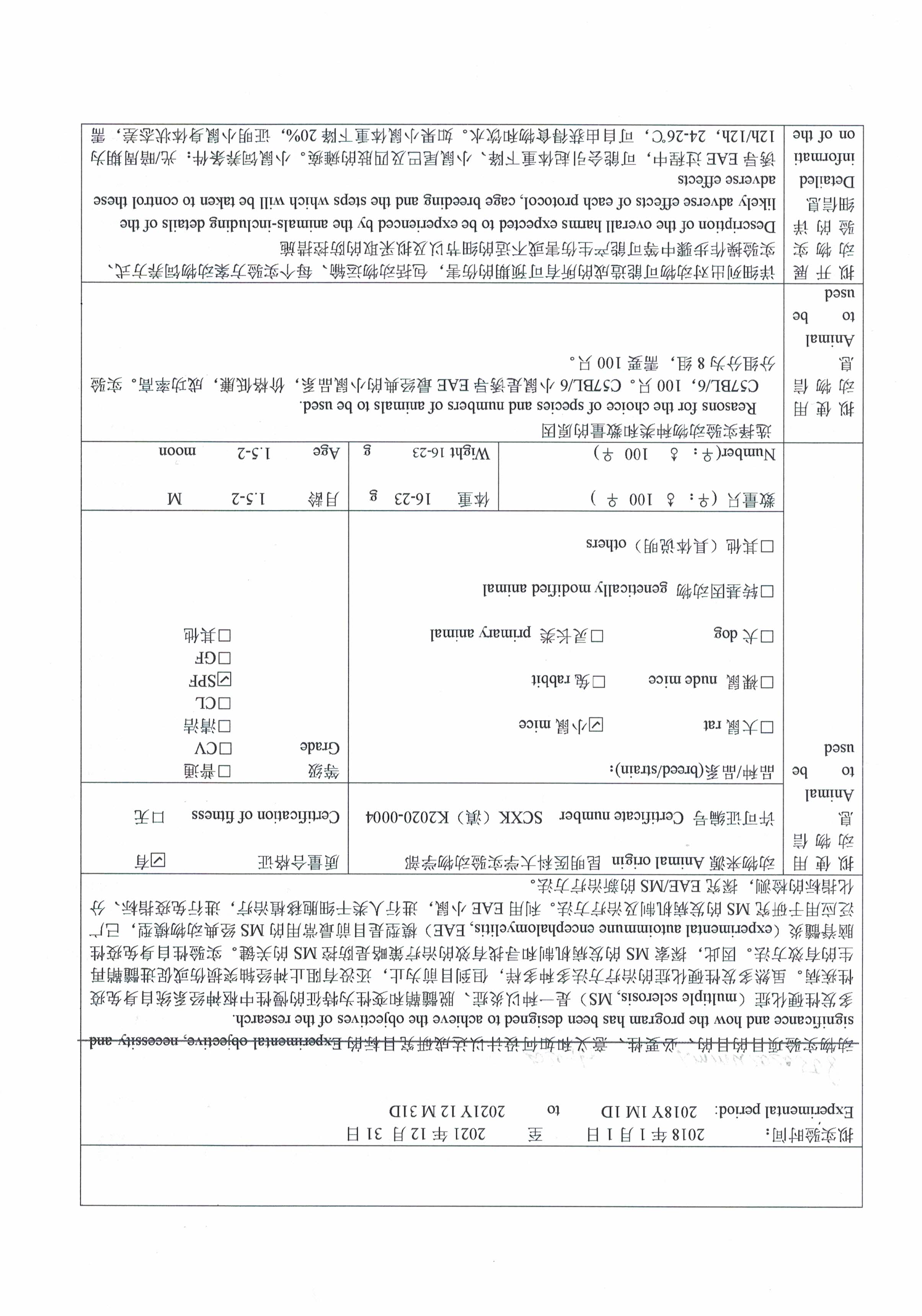

Supplement: S1 File — (ZIP) [file pone.0243014.s002.zip › Ethical approval/CCI_000063.jpg]

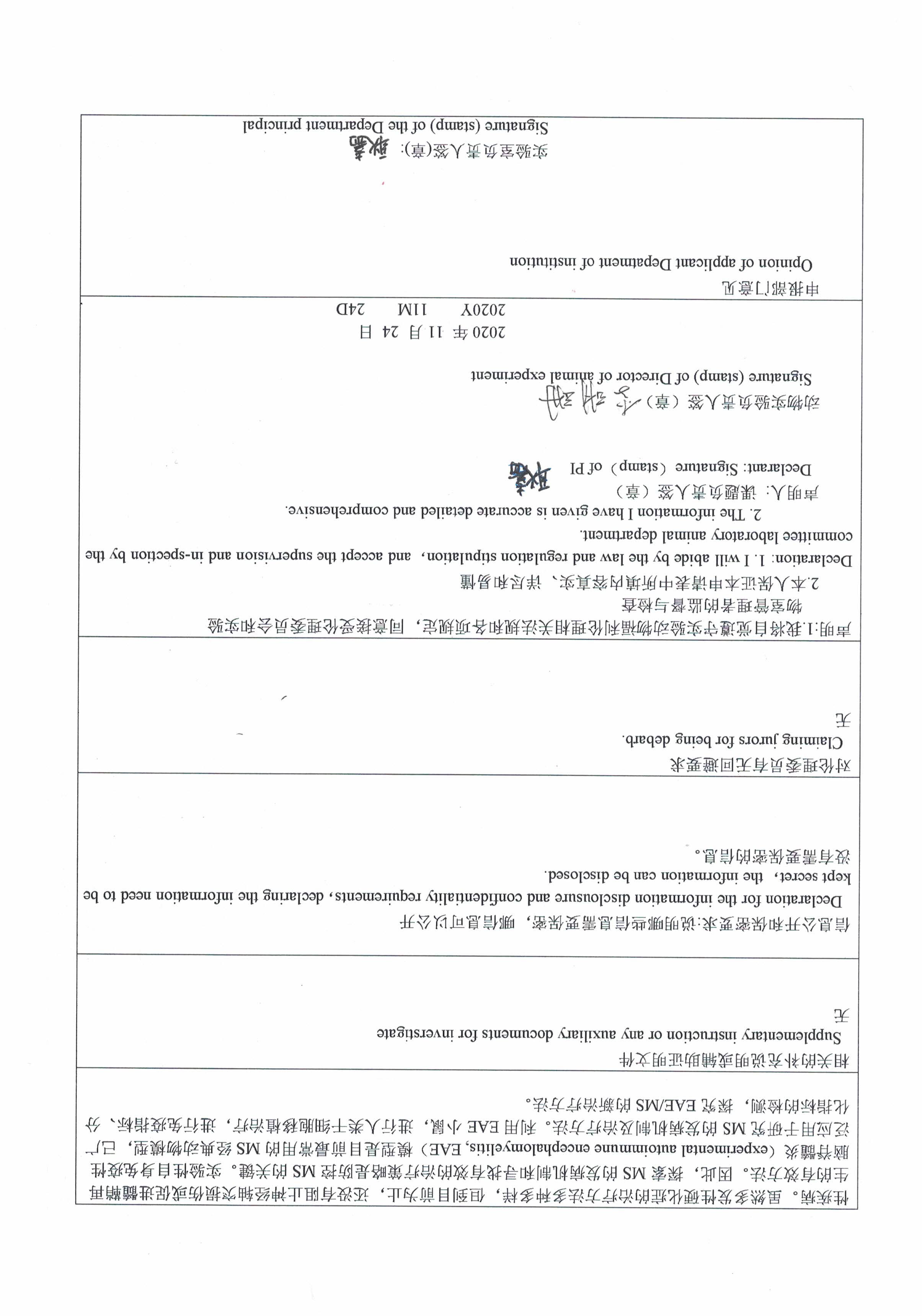

Supplement: S1 File — (ZIP) [file pone.0243014.s002.zip › Ethical approval/CCI_000064.jpg]

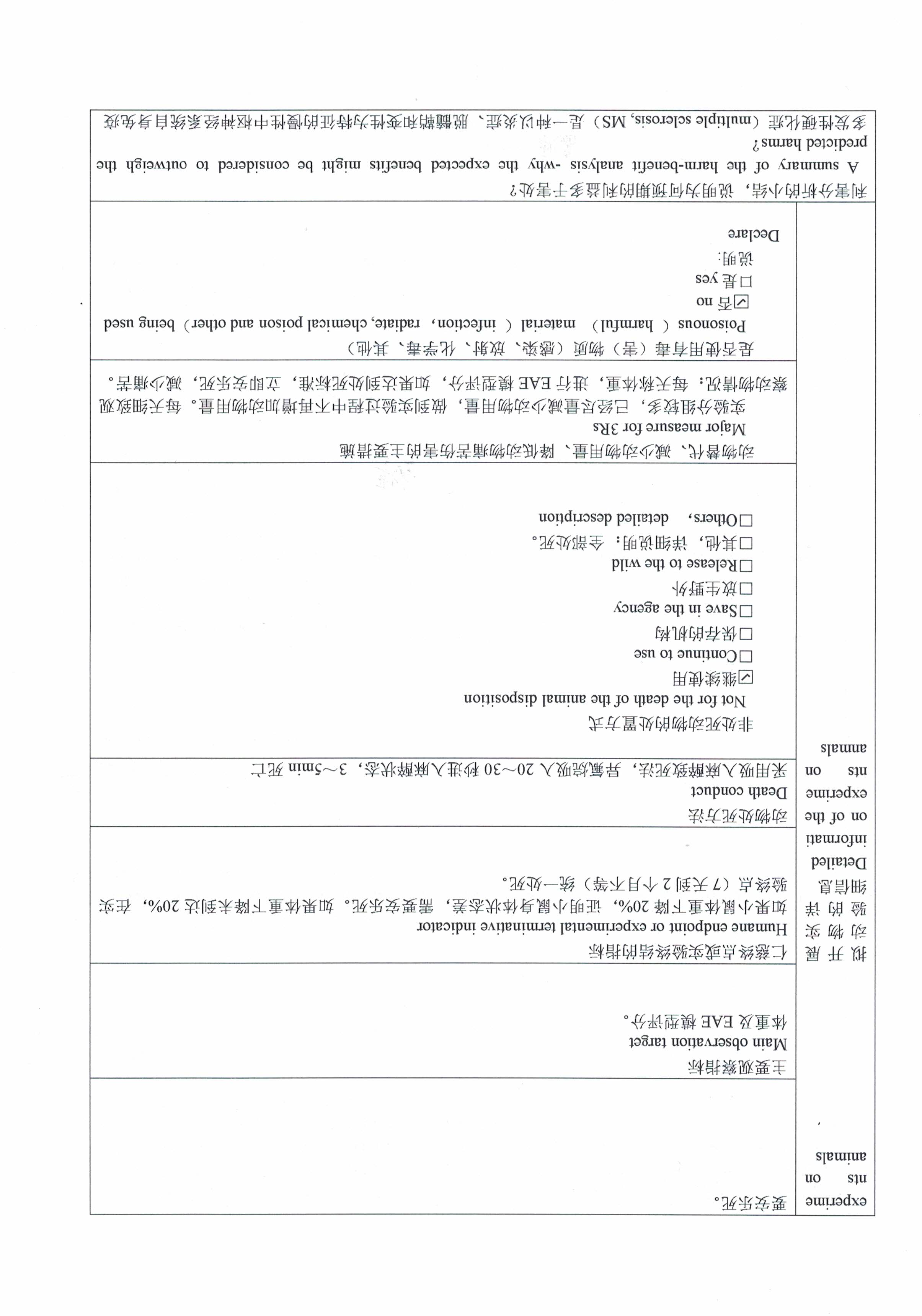

Supplement: S1 File — (ZIP) [file pone.0243014.s002.zip › Ethical approval/CCI_000065.jpg]

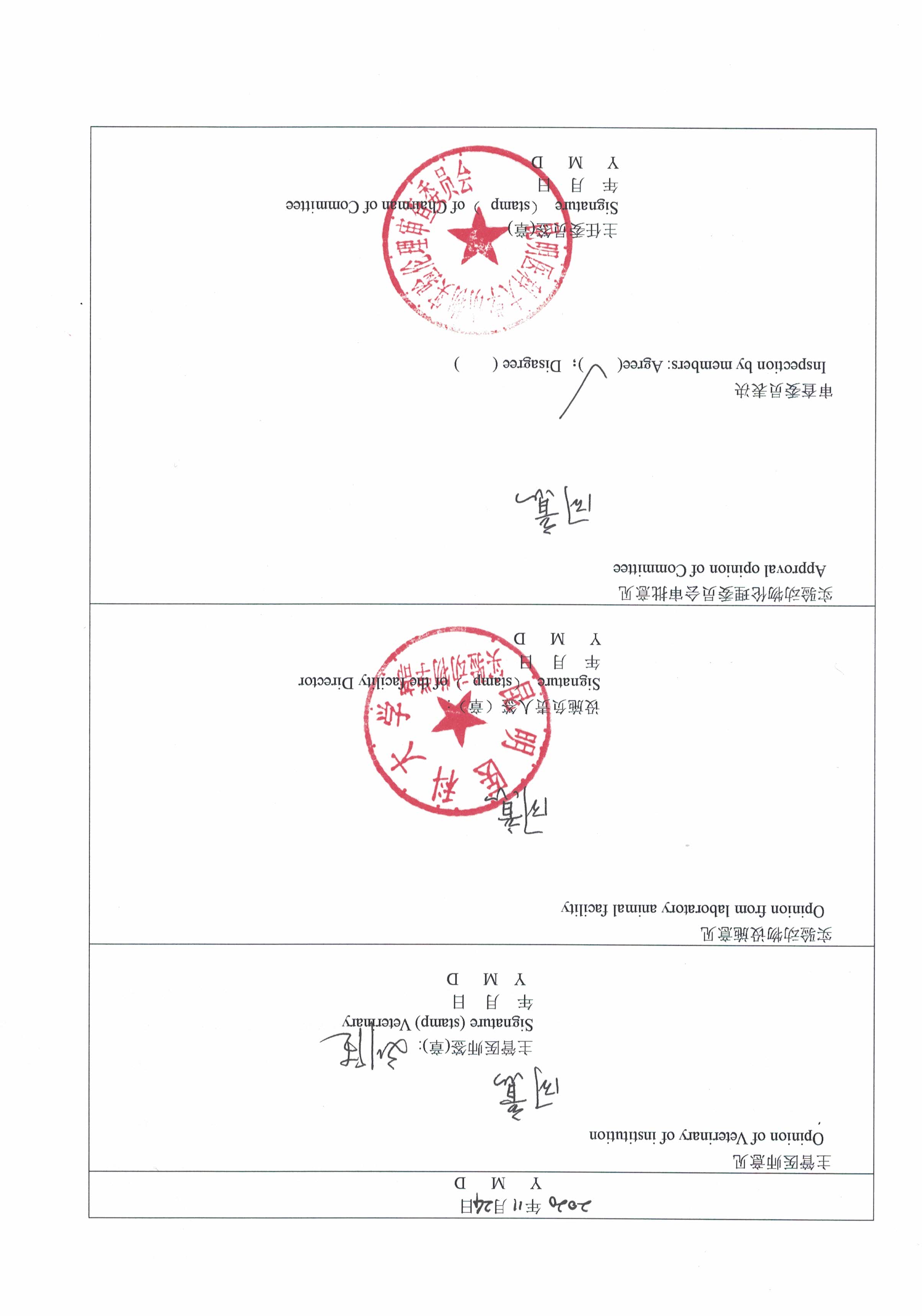

Supplement: S1 File — (ZIP) [file pone.0243014.s002.zip › Ethical approval/CCI_000066.jpg]

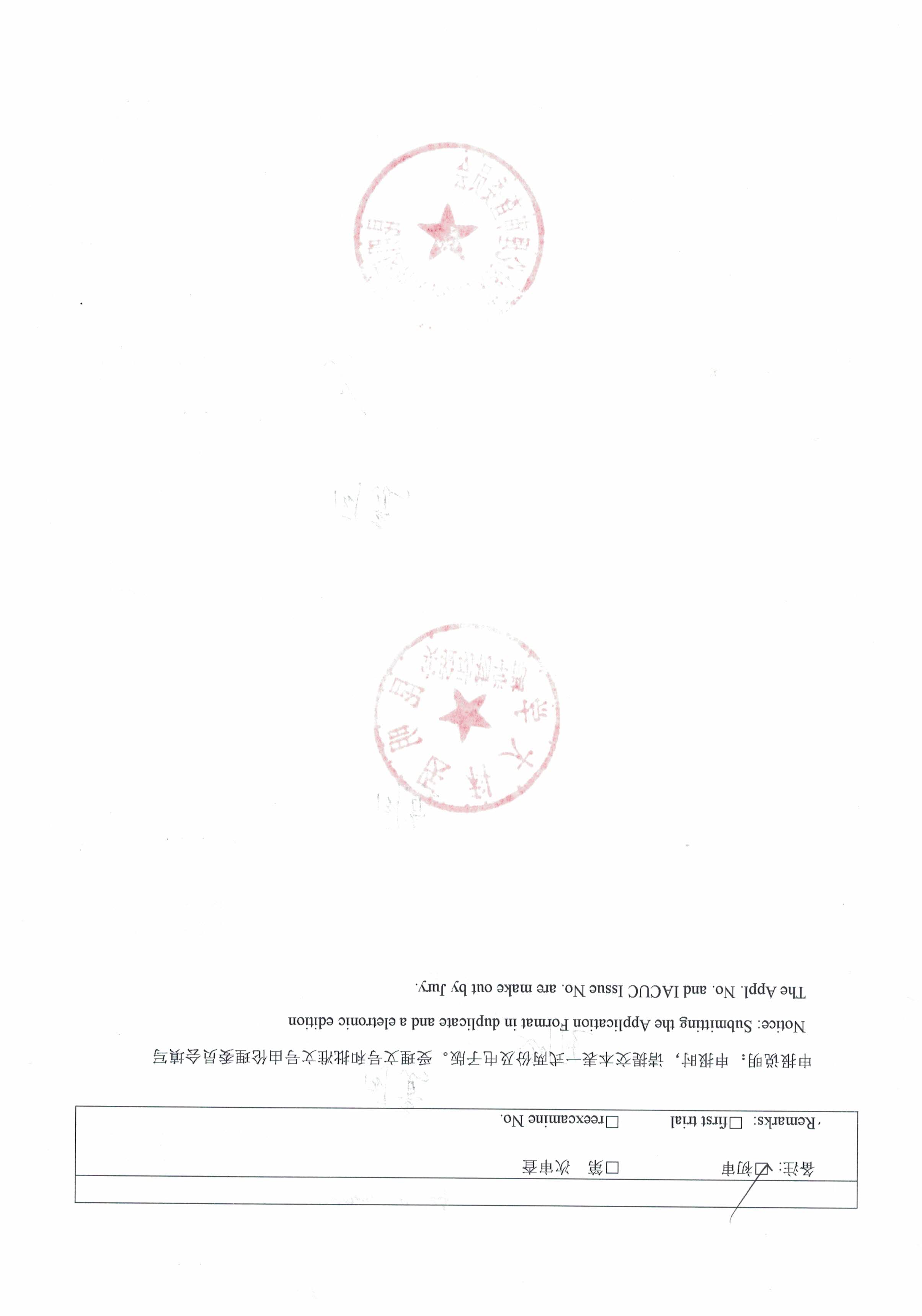

Supplement: S1 File — (ZIP) [file pone.0243014.s002.zip › Ethical approval/CCI_000067.jpg]

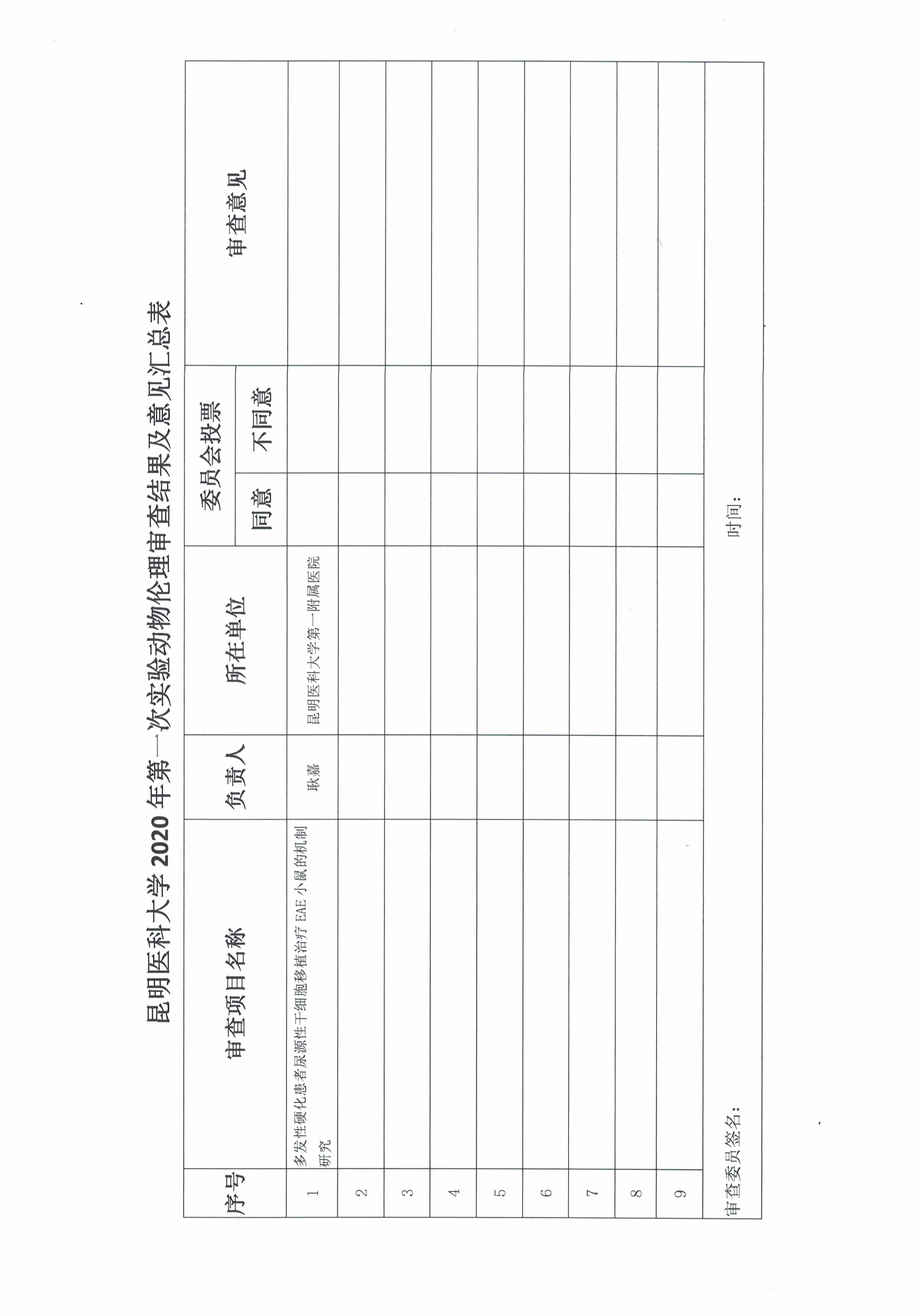

Supplement: S1 File — (ZIP) [file pone.0243014.s002.zip › Ethical approval/CCI_000068.jpg]

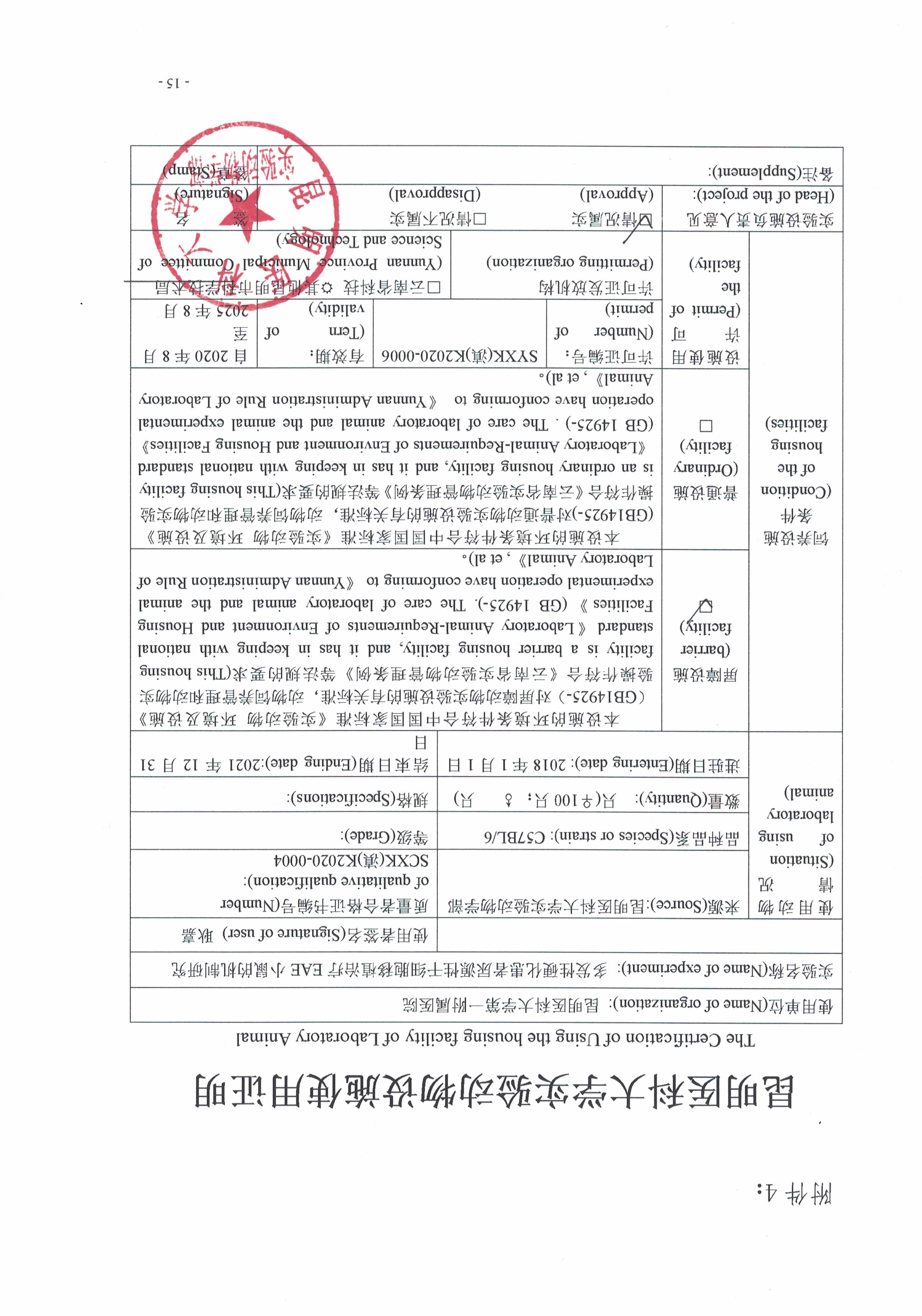

Supplement: S1 File — (ZIP) [file pone.0243014.s002.zip › Ethical approval/CCI_000069.jpg]

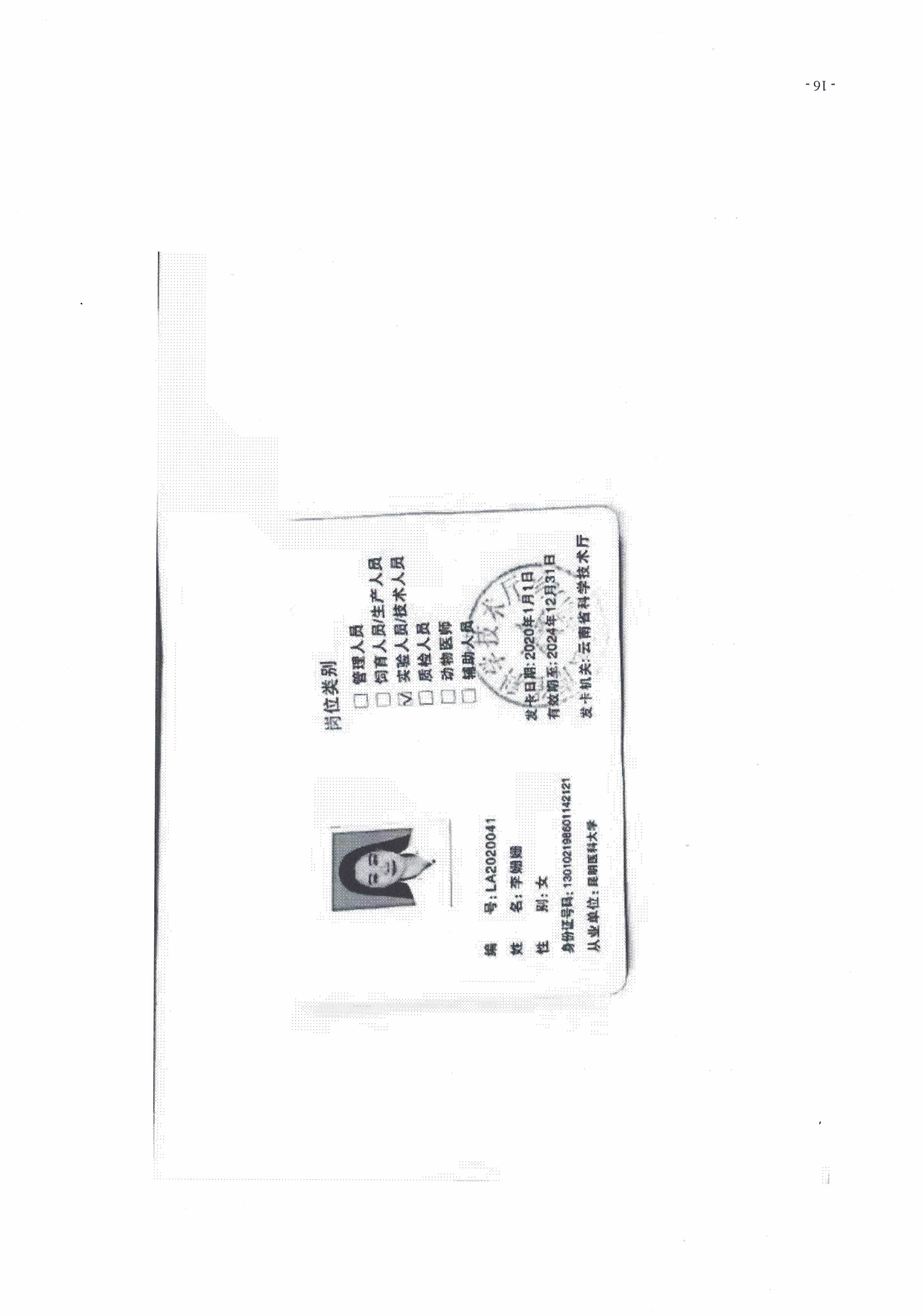

Supplement: S1 File — (ZIP) [file pone.0243014.s002.zip › Ethical approval/CCI_000070.jpg]
