# Supplementary material for: BMSCs differentiated into neurons, astrocytes and oligodendrocytes alleviated the inflammation and demyelination of EAE mice models
Source: PLoS One. 2021 May 13;16(5):e0243014. doi: 10.1371/journal.pone.0243014 (PMC8118321; doi:10.1371/journal.pone.0243014)
Supplement: S1 File — (ZIP) [file pone.0243014.s002.zip › Ethical approval/2013└Ñ├≈╥╜┐╞┤≤╤o╥╜╤o┬╫└φ╬»╘▒╗ß┐╞╤╨┐╬╠Γ┬╫└φ╔≤║╦▒φ.doc]

**昆明医科大学医学伦理委员会**

**科研课题伦理审核表**

| **项目名称**：多发性硬化患者iPS细胞移植治疗EAE小鼠及成神经细胞的机制研究 | | | | | | | | | |
| --- | --- | --- | --- | --- | --- | --- | --- | --- | --- |
| **项目负责人**：耿 嘉 | | | | | | **职称**：讲师 | | | |
| **研究单位**：昆明医科大学第一附属医院 | | | | | | **负责人**：王平 | | | |
| **单位地址**：云南省昆明市西昌路295号 | | | | | | **邮编**：650032 | | | |
| **项目联系人：** 耿 嘉 **电话**：15198999216 **传真**： | | | | | | | | | |
| **电子信箱**：basler777@163.com | | | | | | | | | |
| **合作研究单位**： | | | | | **负责人**： | | | | |
| **联系电话**： | | | **传真**： | | | | **邮编**： | | |
| **研究者**：王文敏 | | **职称**：主任医师 | | **研究者**：李劲涛 | | | | | **职称**：副教授 |
| **研究者：**徐忠 | | **职称**：副主任医师 | | **研究者**：聂志 | | | | | **职称**：讲师 |
| **研究者：**庞爱兰 | | **职称**：讲师 | | **研究者**：陈玲 | | | | | **职称**：博士生 |
| **研究者：**郑颖 | | **职称**：住院医师 | | **研究者：**李玉兰 | | | | | **职称**：硕士生 |
| **研究者：**王立安 | | **职称**：硕士生 | | **研究者：** | | | | | **职称**： |
| **拟研究时间：** 2014 年 1月 1 日至 2016 年 12 月 31 日 | | | | | | | | | |
| **研究课题来源：**□政府 □基金会 □公司 □国际组织 □研究生课题 □其他： | | | | | | | | | |
| **资助者类型：** □政府 □基金会 □公司 □国际组织 □研究生课题 □其他： | | | | | | | | | |
| **资助者名称：**国家自然科学基金委员会 | | | | | | | | | |
| **资助者联系人：** | | | | | | | | | |
| **联系方式：** | | | | | | | | | |
| **请求审查类型：**  □新申请项目 □修订后项目 □延续审查课题 | | | | | | | | | |
| **递交审查资料：**  □实验方案 □知情同意书 □其他资料  （包括：试验用品安全性资料、生产企业资质证明、试验用品提供者的资质证明） | | | | | | | | | |
| 研究内容摘要：  本课题拟制备多发性硬化（MS）的实验性自身免疫性脑脊髓炎（experimental autoimmune encephalomyelitis，EAE）小鼠模型以及MS患者的iPS细胞，并将该细胞体外定向诱导分化成少突胶质细胞、星形胶质细胞和神经元，检测其表面标志物及其电生理特性以及成神经调节因子的变化，探讨其分化机制；同时将iPS细胞植入放疗后的EAE小鼠和对照鼠体内，动态检测其生命期限、运动功能、免疫因子、成神经因子、病理、诱发电位变化和iPS细胞在体内的分布规律，为MS的治疗探索一条新的道路。 | | | | | | | | | |
| **保密要点**：有关受试者的医学记录和研究资料都是保密的。研究结果发表时，与受试者有关的信息资料不会被公开。 | | | | | | | | | |
| **主要研究者资格评价**：   - 课题负责人具备进行本项研究的资格 - 课题负责人不具备进行本项研究的资格   **研究方案评价：**   - 课题研究方案符合医学伦理规范 - 课题研究方案不符合医学伦理规范   **知情同意书评价：**   - 知情同意书符合医学伦理规范 - 知情同意书不符合医学伦理规范 | | | | | | | | | |
| **结**  **论** | **1. 同意** | | | | | | |  | |
|  | **2. 作必要修改后同意** | | | | | | |  | |
|  | **3. 不同意** | | | | | | |  | |
| **伦理委员会审查意见：**  **昆明医学院医学伦理委员会**  **年 月 日** | | | | | | | | | |

填表说明：1、申请日期请填写拟交申请日期。

2、申请书中方格可在文字输入打印后，在选中的项目前用钢笔画√。

3、联系人为：本研究项目的联系人及电话。

4、研究者包括合作研究单位的人员。

5、请求审查类型中：延续审查课题为：一项课题需第二次审查的课题。

6、送交审查资料包括：申请书、试验方案、知情同意书；如为人体用品还需按其他资料项目要求提交资料。
